# Evaluation of Cognitive Functions in Patients with Acute Coronary Syndrome Undergoing Percutaneous Coronary Intervention: A Prospective Pilot Study

**DOI:** 10.3390/diagnostics14141492

**Published:** 2024-07-11

**Authors:** Gozde Baran, Suleyman Sezai Yildiz, Ozge Gonul Oner, Ahmet Gurdal, Kudret Keskin, Serhat Sigirci, Kadriye Orta Kilickesmez, Gulsen Babacan Yildiz

**Affiliations:** 1 Department of Neurology, Sancaktepe Sehit Prof. Dr. Ilhan Varank Research and Training Hospital, 34785 Istanbul, Turkey; 2 Department of Cardiology, University of Health Sciences, SBÜ Prof. Dr. Cemil Tascioglu City Hospital, 34384 Istanbul, Turkey; 3 Department of Neurology, Goztepe Prof. Dr. Suleyman Yalcin City Hospital, 34722 Istanbul, Turkey; ozge.gonl@gmail.com; 4 Department of Cardiology, University of Health Sciences, Sisli Hamidiye Etfal Training and Research Hospital, 34396 Istanbul, Turkey; 5 Private Consultant, 34365 Istanbul, Turkey; 6 Department of Cardiology, Liv Hospital, 34340 Istanbul, Turkey; 7 Department of Neurology, Istanbul Research and Training Hospital, 34098 Istanbul, Turkey

**Keywords:** cognitive function, structural MRI, cognitive test, acute coronary syndrome

## Abstract

Purpose: It is not clear whether cognitive functions are impaired in young patients with acute coronary syndrome (ACS). This study aims to detect whether or not there is cognitive impairment and cerebral changes in young patients with ACS undergoing percutaneous coronary intervention (PCI). Patients and Methods: All 50 patients with ACS who were treated with primary PCI were eligible for this prospective study. All participants had normal cognitive function before ACS. Brain magnetic resonance imaging (MRI) was performed to quantify changes in brain white and gray matter. Cognitive functions (CFs) were evaluated by seven cognitive tests. Patients were categorized by MRI findings and test scores were compared from the first day to after the first month. Results: We determined 25 patients with impaired CFs on the first day. After the first month, we identified 18 patients with transient impaired CFs. No structural difference was observed between impaired CF and normal CF. While 25 patients had a score of 1 according to Fazekas, 10 patients had a score of 1 according to MTLA. While the mean Stroop test completion time and Stroop test error rate scores were significantly higher on the first day than after the first month in the Fazekas+ group (*p* = 0.003, *p* < 0.001, respectively), other cognitive test scores—except clock drawing test, digital span forwards, and backwards—were significantly lower on the first day compared to after the first month in the Fazekas+ group (*p* < 0.05). Conclusions: Patients with ACS have transient impairment in cognitive functions. Acute coronary syndrome is not associated with structural changes in the brain.

## 1. Introduction

Acute coronary syndrome (ACS), which consists of ST-segment elevation myocardial infarction (MI), non-ST-segment elevation MI, and unstable angina, is a very common and life-threatening health problem [1]. Cognitive impairment without dementia (CIWD) is characterized by cognitive decline that is confirmed by family members and proven in neuropsychological tests without affecting daily activities [2]. Cardiovascular risk factors such as hypertension, diabetes, and hyperlipidemia have been reported as possible risk factors for CIWD. Another proposed risk factor is lower brain size and neuron density [3]. Although up to 35% of patients with ACS have cognitive impairment, the exact relationship in terms of cause and effect between cognitive impairment and ACS is unclear. The development of cognitive impairment due to ACS may have several implications, such as the patient’s ability to change lifestyle habits and adhere to new medication regimens to prevent further events. Moreover, the impact of non-cardiac comorbid conditions such as dementia or CIWD on further deterioration of cognitive functions after ACS is not fully elucidated [4]. Therefore, an accurate assessment of cognitive impairment in patients with ACS remains to be established.

In our study, we aimed to investigate (1) whether there is evidence of short-term cognitive impairment by cognitive tests, (2) cardiovascular and sociodemographic risk factors affecting cognitive functions in the short term, and (3) the relationship between white matter lesions (WMLs) and/or hippocampal volume loss using structural MRI in patients with ACS undergoing PCI.

## 2. Materials and Methods

This prospective pilot study included patients aged between 35 and 60 years old who presented with ST-segment elevation myocardial infarction (STEMI), non-ST-segment elevation myocardial infarction (NSTEMI), or unstable angina (UA) between April 2018 and July 2018. The diagnosis of STEMI and NSTEMI was made according to the third universal definition of myocardial infarction [5]. Unstable angina (USAP) was defined as the absence of cardiomyocyte necrosis. Since cognitive status is affected by neurodegenerative processes, older patients were not recruited. We also excluded patients with known heart failure, atrial fibrillation, neurological disorders, psychotic disorders, history of major depression, thyroid dysfunction, and cancer, liver, and renal failure. Additionally, patients who had hearing and vision problems, were illiterate, or had a fear of MRI were considered ineligible. Age, sex, education level, family history of dementia, consanguineous marriage, smoking status and alcohol use, electrocardiography, physical exam, laboratory tests, and MRI examinations were carefully recorded for each case. Cardiovascular risk factors, prior medical history, and medication were also recorded. All subjects underwent coronary stenting following coronary angiography and also received medical treatment. The left ventricular ejection fraction was evaluated by transthoracic echocardiography. A complete neurological exam was performed by a neurologist (GB). The study was conducted according to the recommendations of the Declaration of Helsinki on Biomedical Research Involving Human Subjects and the Good Clinical Practice Guidelines. The study protocol was approved by the ethics committee at our institute, and informed consent was obtained in writing from each subject before the study.

### 2.1. Cognitive Evaluation

Neuropsychological tests were administered and scored by a specialist neurologist (GB) on the first day of and one month after PCI. All cognitive tests used in this study were modified to fit the local culture and language. General cognitive functions were evaluated with the Mini-Mental State Exam (MMSE) [6]. The clock drawing test (CDT) was used to evaluate planning, ranking, and abstract thinking skills using visual–motor functions [7]. The digit span forwards and backwards tests were used to assess attention and working memory functions [8]. The Boston Naming Test (BNT) was used to investigate the patient’s language cognitive domain [9]. The phonemic fluency test, which involved the production of words beginning with specified letters K, A, and S, was used to evaluate working memory. The categorical (animal) fluency test was used to measure semantic fluency, semantic judgment, and semantic retrieval [10]. The Stroop test was used to evaluate the cognitive function of inhibitory control [11].

Cognitive dysfunctions without dementia were diagnosed clinically using MCI-revised criteria [12]. The diagnosis of cognitive impairment was based on the clinical exclusion of mild cognitive impairment and a decline in at least one of the cognitive tests. The depression and anxiety status of the patients were measured using the Beck Depression Inventory (BDI) and Beck Anxiety Inventory (BAI) [13,14].

### 2.2. Magnetic Resonance Imaging

All MR imaging examinations were performed with a 1.5 T MR system (Magnetom; Avanto, Siemens, Erlangen, Germany) using an 8-channel head coil on the first day after PCI. Coronal T1-weighted images were acquired with TR, 490 ms; TE, 9.7 ms; FOV, 23; and section thickness, 5 mm with a 1.5 mm gap. Axial T2-weighted images were acquired with TR, 4000 ms; TE, 85 ms; FOV, 23; and section thickness, 5 mm with a 2 mm gap. Axial fluid-attenuated inversion-recovery (FLAIR) images were acquired with TR, 8100 ms; TE, 88 ms; FOV, 23; and section thickness, 5 mm with a 2 mm gap.

Temporal lobe atrophy was evaluated by the Medial Temporal Lobe Atrophy (MTLA) Score in the coronal section of T1-weighted sequences based on the measurement of the width of the choroid fissure and temporal horn and the height of the hippocampal formation. Medial Temporal Lobe Atrophy was scored on a range from 0–4: 0, no atrophy; 1, only widening of the choroid fissure (minimal atrophy); 2, also widening of the temporal horn of the lateral ventricle (moderate atrophy); 3, moderate loss of hippocampal volume (decrease in height—severe atrophy); 4, severe volume loss of the hippocampus (marked atrophy) [15]. In FLAIR sequences, periventricular and deep white matter lesions were assessed with Fazekas [16]. It is particularly known that deep white matter scoring is useful in assessing possible dementia patients. Deep WMLs were scored on a range from 0–3: 0, no lesion; 1, punctate lesions; 2, fused lesions, and 3, large fused lesions. Periventricular, deep white matter hyperintensities, and MTLA scores were independently rated by two researchers who were not aware of patient clinical information (EU, GBY). Based on MRI findings, patients were stratified by MTLA scores as MTLA score 0 group (MTLA−) and MTLA score ≥ 1 group (MTLA+) and by deep WMLs as Fazekas score 0 (Fazekas−) and Fazekas score ≥ 1 group (Fazekas+), and cognitive test results were compared between these groups.

### 2.3. Statistical Analysis

The data are presented as the mean, standard deviation (SD), and percentages. All analyses were performed using IBM SPSS Statistics, V.20.0 (Armonk, NY, USA: IBM Corp.). Changes in neurological scales across different times between the 1st day and 1st month were analyzed by the Wilcoxon Signed Rank Test within groups. We used Fisher’s exact test and the chi-square test to assess the association between two qualitative variables. Longitudinal Analysis of Cognitive Scores was performed to examine the changes between Day 1 and Day 30. For each cognitive test, the change in scores from Day 1 to Day 30 was calculated for both Fazekas− and Fazekas+ groups. To compare the change in cognitive test scores between the Fazekas− and Fazekas+ groups over a period from Day 1 to Day 30, the independent *t*-test was used to compare the means of two independent groups (Fazekas− and Fazekas+), and the Mann–Whitney U test was used to compare differences between two independent non-parametric groups. For each cognitive test, the change in scores from Day 1 to Day 30 was calculated for both MTLA− and MTLA+ groups. To compare the change in cognitive test scores between the MTLA− (Medial Temporal Lobe Atrophy negative) and MTLA+ (Medial Temporal Lobe Atrophy positive) groups over a period from Day 1 to Day 30, the independent *t*-test was used to compare the means of two independent groups (MTLA− and MTLA+), and the Mann–Whitney U test was used to compare differences between two independent non-parametric groups. *p* < 0.05 was considered statistically significant.

## 3. Results

According to the cognitive function tests on the first day, 22 patients (mean age: 48.04 ± 7.76, 52% male) had impaired CF, and 28 patients (mean age: 51.70 ± 6.46, 56% male) had normal CF. Table 1 shows the demographics, laboratory findings, clinical features, and cardiac risk profiles of all cases. There were no significant differences among the groups with respect to age, right-handedness, body mass index (BMI), marriage, educational level, history of family dementia, systolic and diastolic blood pressure, heart rate, and smoking status (*p* > 0.05 for all; Table 1). Creatinine, low-density lipoprotein cholesterol, total cholesterol, estimated glomerular filtration rate, and fasting glucose of each group were similar (*p* > 0.05 for all; Table 1). The development of atrial fibrillation, left ventricular ejection fraction, vitamin B12, folic acid, and vitamin D values were similar between groups. Serum cardiac troponin levels were significantly higher in the ICF group compared with the NCF group (47.5 ± 26.2, 34.8 ± 22.0, *p* = 0.01, respectively; Table 1). The average hospital stay was 5.3 days for the ICF group and 5.0 days for the NCF group.

**Figure 1:**
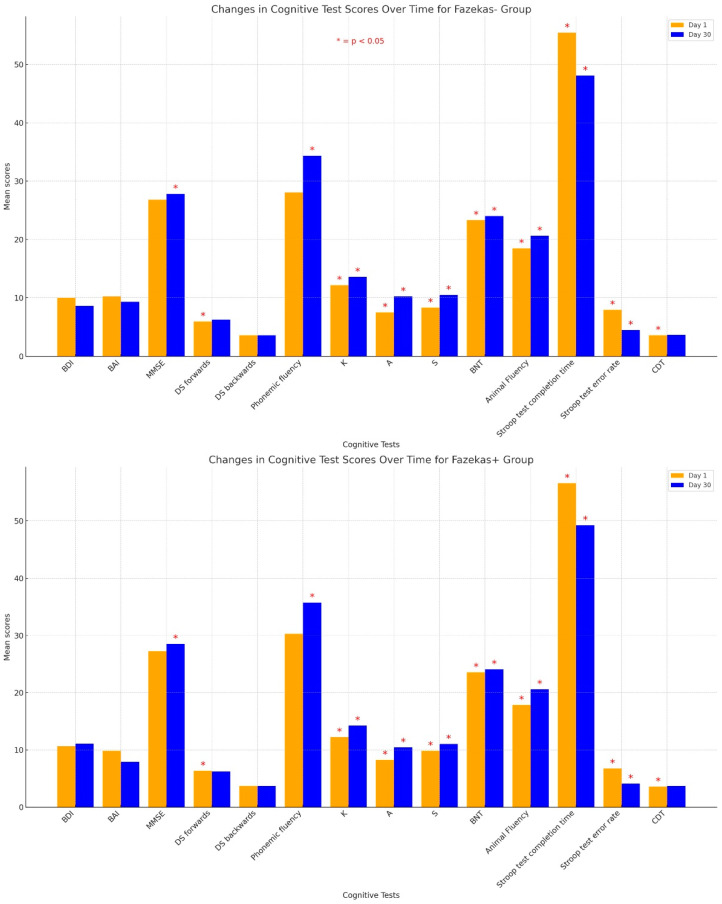
Cognitive test score changes over time in Fazekas groups.

Table 2 lists the mean neurocognitive test scores and the mean BDI and BAI scores on the first day and on the thirtieth day according to Fazekas scores of the study population. None of the cases had documented anti-depressive or anti-anxiety medication. The mean BDI and BAI scores were similar and not statistically significant among the groups on the first day and on the thirtieth day (*p* > 0.05). Although the BAI score especially decreased in the first month in both groups, this was not statistically significant. The mean MMSE, DS forwards, phonemic fluency (K, A, and S letters), animal fluency, and BNT scores were significantly lower on the first day compared to the thirtieth day in the Fazekas 0 group (*p* = 0.001, *p* = 0.021, *p* < 0.001 [*p* = 0.029 for K letter, *p* = 0.001 for A letter, and *p* = 0.001 for S letter], *p* < 0.001, and *p* = 0.025, respectively). The mean Stroop test completion time and Stroop test error rate scores were significantly higher on the first day than on the thirtieth day in the Fazekas 0 group (*p* = 0.003 and *p* < 0.001, respectively). The mean DS backwards and CDT scores were not statistically significant (*p* = 1.000 and *p* = 0.317, respectively). The mean MMSE, phonemic fluency (K, A, and S letters), BNT, and animal fluency scores were significantly lower on the first day than on the thirtieth day in the Fazekas 1 group (*p* = 0.003, *p* < 0.001 [*p* = 0.018 for K letter, *p* < 0.001 for A letter, and *p* = 0.037 for S letter], *p* < 0.001, *p* = 0.007, and *p* = 0.004, respectively). The mean DS forwards and DS backwards scores were not statistically significant (*p* = 0.768 and *p* = 0.916, respectively). The Stroop test completion time and Stroop test error rate scores were significantly higher on the first day than on the thirtieth day (*p* = 0.001 and *p* < 0.001, respectively). Cognitive test score changes over time in Fazekas groups are shown in Figure 1.

**Figure 2:**
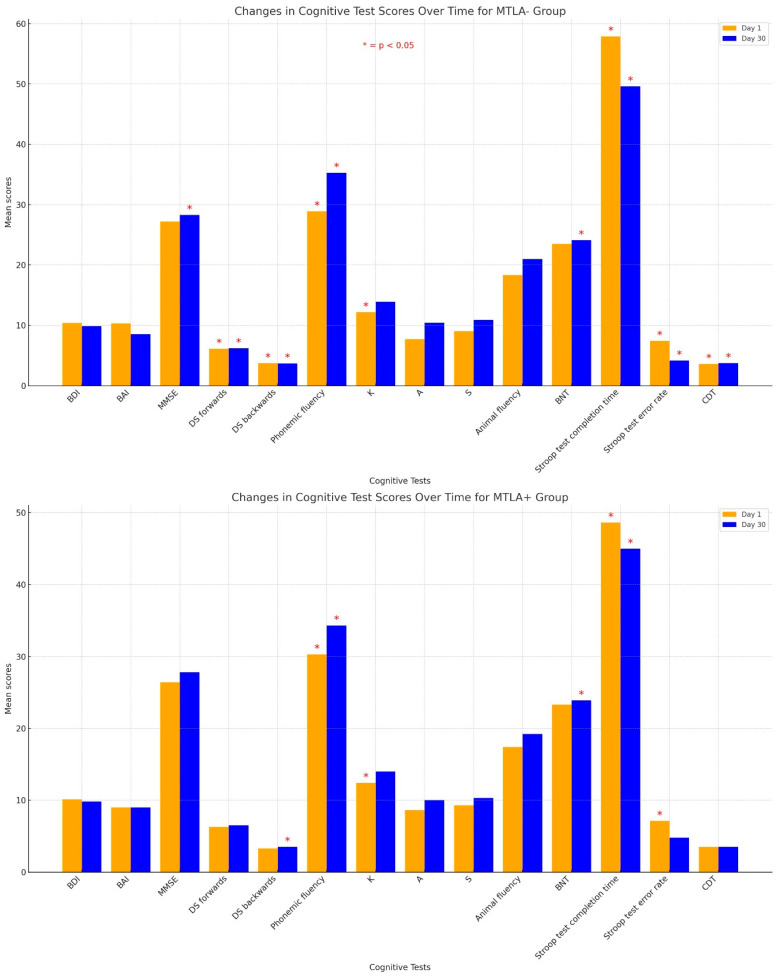
Cognitive test score changes over time in MTLA groups.

Table 3 lists the mean neurocognitive test scores and the mean BDI and BAI scores on the first day and on the thirtieth day in patients with and without MTLA. The mean BDI score was not statistically significant among the groups on the first day and the thirtieth day (*p* > 0.05). The mean BAI score was significantly lower on the thirtieth day than on the first day in the MTLA− group (*p* = 0.005). The mean MMSE, phonemic fluency (K, A, and S letters), and BNT scores were significantly lower on the first day than on the thirtieth day in the MTLA− group (*p* < 0.001, *p* = 0.005 [*p* = 0.002 for K letter, *p* = 0.043 for A letter, and *p* < 0.001 for S letter], and *p* = 0.003, respectively). The mean Stroop test completion time and Stroop test error rate scores were significantly higher on the first day than on the thirtieth day in the MTLA− group (*p* = 0.03 and *p* = 0.02, respectively). The mean DS forwards, DS backwards, animal fluency, and CDT scores were not statistically significant (*p* = 0.169, *p* = 0.870, *p* = 0.118, and *p* = 0.06, respectively). The mean BDI and BAI scores were not statistically significant among the groups in the MTLA+ group (*p* = 0.765 and *p* = 0.887, respectively). The mean MMSE and phonemic fluency test scores were significantly lower on the first day than on the thirtieth day in the MTLA+ group (*p* = 0.03 and *p* = 0.024, respectively). The mean DS forwards, DS backwards, phonemic fluency (for K, A, and S letters), animal fluency, BNT, and CDT scores were not statistically significant (*p* > 0.05 for all). Cognitive test score changes over time in MTLA groups are shown in Figure 2.

**Figure 3:**
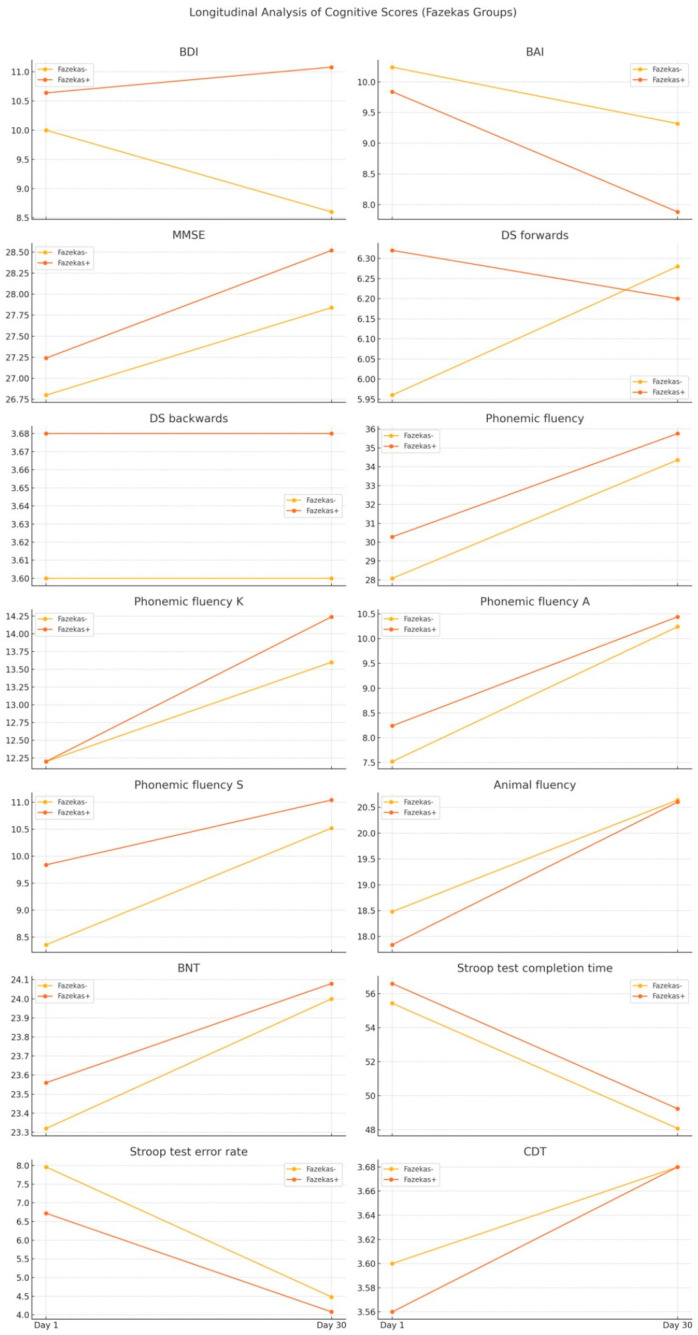
Longitudinal analysis of the cognitive scores for Fazekas+ and Fazekas− patients.

**Figure 4:**
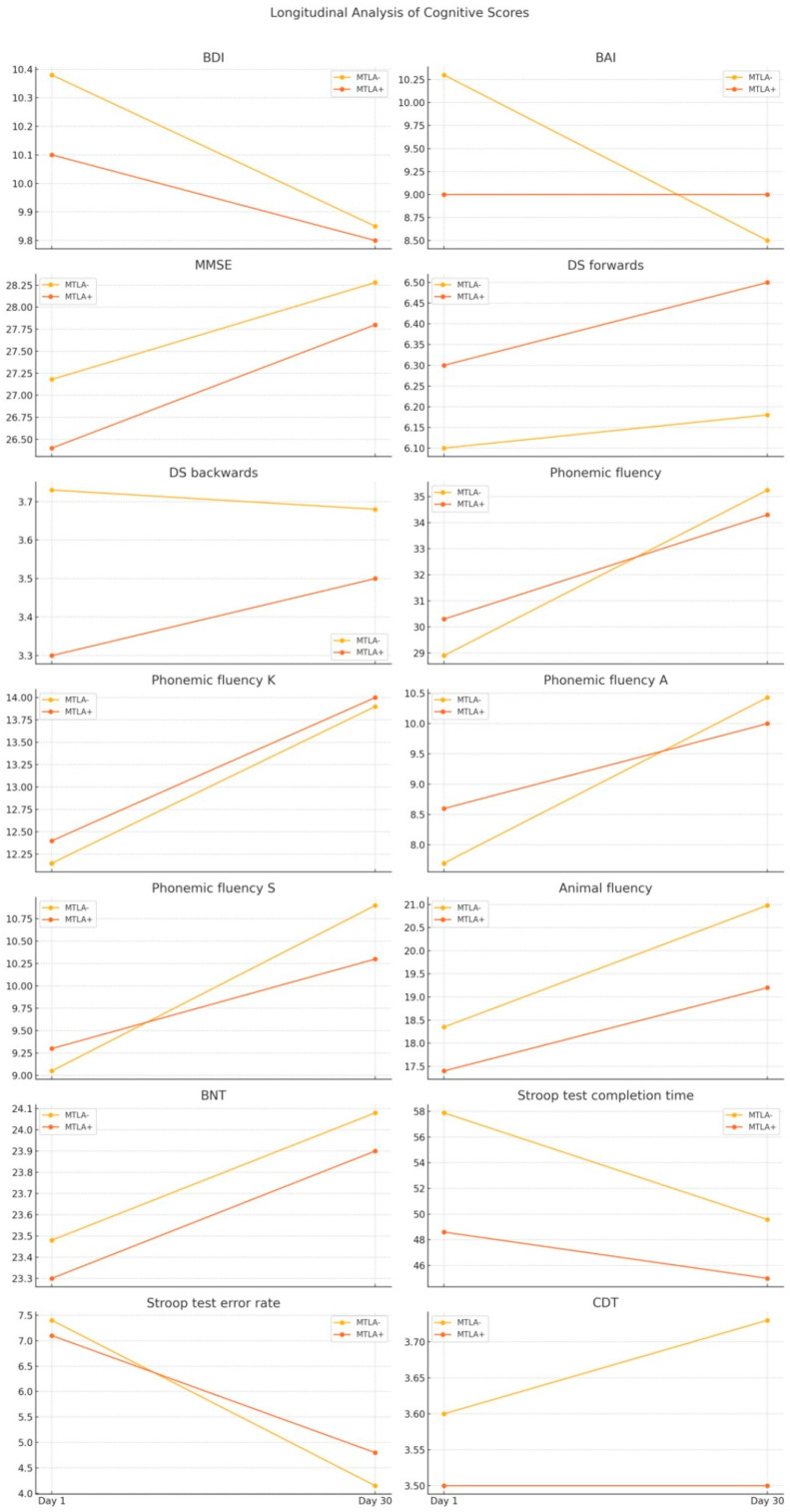
Longitudinal analysis of the cognitive scores for MTLA+ and MTLA− patients.

Longitudinal analysis of cognitive scores was performed to examine the changes between Day 1 and Day 30. Comparison of the change in cognitive test scores between the Fazekas− and Fazekas+ groups over a period from Day 1 to Day 30 revealed no significant differences (Table 4). The *p*-values obtained from both the independent *t*-test and the Mann–Whitney U test indicate that there are no statistically significant differences in the changes in cognitive test scores between the Fazekas− and Fazekas+ groups from Day 1 to Day 30. All *p*-values are greater than 0.05, suggesting that the changes in cognitive function over time are similar in both groups, regardless of the presence of Fazekas lesions. Longitudinal analysis of cognitive scores for Fazekas+ and Fazekas− patients is demonstrated in Figure 3. Comparison of the change in cognitive test scores between the MTLA− (Medial Temporal Lobe Atrophy negative) and MTLA+ (Medial Temporal Lobe Atrophy positive) groups over a period from Day 1 to Day 30 revealed no significant differences either. The *p*-values obtained from both the independent *t*-test and the Mann–Whitney U test indicate that there are no statistically significant differences in the changes in cognitive test scores between the MTLA− and MTLA+ groups from Day 1 to Day 30 (Table 5). All *p*-values are greater than 0.05, suggesting that the changes in cognitive function over time are similar in both groups, regardless of the presence of Medial Temporal Lobe Atrophy. Longitudinal analysis of cognitive scores for MTLA+ and MTLA− patients is demonstrated in Figure 4.

## 4. Discussion

The primary aim of this study was to determine whether or not there was post-ACS cognitive dysfunction, and if present, to identify the subdomains that were primarily affected. None of our patients had prior dementia diagnosed before ACS, and we did not detect acute dementia after ACS. Although 22 patients had impaired cognitive functions, 72% of them reached normal cognitive function at follow-up. There were no structural differences between patients with impaired and normal cognitive function on the first day of ACS. While half of the patients with ACS had a dotted lesion according to the Fazekas score, only one fourth showed minimal atrophy with the MTLA score. Traditional cardiovascular risk factors were not associated with cognitive impairment in patients with ACS. To the best of our knowledge, the present study is the first one using brain imaging and cognitive tests to investigate the cognitive functions in patients with ACS.

CIWD is a subclinical condition that sometimes the patient does not even notice, and for this reason, it is easily overlooked. Although there are several studies reporting some impairment in cognitive functions after coronary bypass surgery, the studies on whether cognitive functions are affected after ACS, and which subdomain is affected, are very limited [17]. In our study, we used comprehensive neuropsychological assessment and tests that evaluated all subdomains of cognitive functions. We performed all of the tests on both post-ACS Day 1 and Day 30 and found deterioration. Gharacholo et al. reported that more than half of the post-ACS patients had CIWD [18]. In our study, we showed that total MMSE scores at Day 30 were significantly improved compared to those on Day 1. In addition, we detected improvements in phonemic fluency and BNT. We compared cognitive impairment in terms of sociodemographic characteristics and cardiac and biochemical parameters. However, there was no significant difference in neuropsychological assessments performed on either Day 1 or Day 30 in terms of these parameters. Stress, smoking, age, sex, and BMI, which are established risk factors for cardiovascular diseases, were also evaluated regarding their effects on cognitive functions in our study.

We also examined if our patients had depression or anxiety and their possible effects on cognitive functioning. Studies in the literature have reported depression rates ranging from 35% to 45% after cardiovascular events [19,20]. We detected depression in 40% and anxiety in 50% of our patients. While there was no significant difference between neuropsychological tests of patients with and without depression, patients with anxiety had worse digit forward span scores, which provide information about attention and working memory, compared to those of their counterparts without anxiety. Depression scores of patients did not change after ACS, whereas anxiety scores had significantly improved at Day 30.

There is significant evidence that smoking is a serious risk factor for ACS, yet its effects on cognitive functions remain unclear [21]. Nooyens et al. found that smokers performed worse in all cognitive functions compared to non-smokers in a large healthy cohort [22]. In our study, 38% of the patients (range: 10–120 pack-years) were smokers. There was no significant difference in cognitive functions between smokers and non-smokers. Approximately 3% to 10% of all ACS patients are 45 years of age and younger [23]. However, this rate was slightly higher in our patients (28%). We attributed this to the fact that our patients had to be literate. Numerous studies have reported deterioration in many subdomains of cognitive function with aging [24,25]. However, the oldest patient in our study was 64 years old, and we did not detect any significant difference in cognitive function or neuroimaging between younger patients and those > 45 years old. In addition, men and women did not differ in terms of cognitive functions in our study. The relationship between BMI and cognitive functions is controversial. While some studies reported that obesity and high BMI were associated with impaired cognition, several studies and meta-analyses reported improved cognitive functions among individuals with high BMI [26,27]. Seventy percent of our patients were overweight (BMI > 25) and 10% were obese. Patients with normal and high BMI did not significantly differ in terms of cognitive functions.

Many studies have reported that lesions in the white matter lead to both dementia and cognitive impairment [28,29,30]. WMLs have been reported in healthy community-based studies as 50.9% in healthy persons between the ages of 44–48 and 95% in healthy persons between the ages of 60–90 years [30]. We used Fazekas classification to evaluate WML and found Fazekas 1 in 28.6% of the ≤45-year-old group and in 58% of the >45-year-old group. We speculated that the injury apparent in coronary arteries might also have occurred in cranial vessels. We also investigated the relationship of Fazekas stage to CIWD within the whole group but did not find any significant difference. Medial Temporal Lobe Atrophy was measured using the scale developed by Scheltens et al. [15]. According to this scale, scores of 2 and above are considered abnormal for those under 75 years. While 80% of our patients had a score of 0 on this scale, the rest had a score of 1. There were no scores of 2 or higher. There was no difference in cognitive functions between those obtaining scores of 0 and 1. The MTLA score was 1 point in 7.1% of the patients in the ≤45-year-old group and 25% of the patients in the >45-year-old group, with no statistically significant difference existing. It is considered important that MTLA+ patients be followed up in terms of potential progression of their existing atrophy in the long term.

## 5. Study Limitations

Our study had several limitations. First, illiterate and elderly patients were not included in the study. Secondly, there was no control group to compare the data obtained from the patients. Thirdly, the effect of the drugs used by the patients on cognitive functions was not evaluated. Fourthly, a functional MRI could not be performed. Finally, although the patients in our study were tested in a relatively low-stress clinic setting, patients with ACS who underwent PCI most likely experienced emotional stress.

## 6. Conclusions

We detected transient cognitive impairment in half of the patients with ACS undergoing PCI. In addition, none of these patients had a structural defect in the brain. This pilot study underlined the importance that patients with ACS are at risk for cognitive impairment. It is considered that routine clinical management of post-ACS strategy should incorporate CIWD-focused follow-up and treatment as part of a multidisciplinary approach. However, further studies are needed to better understand the pathophysiology of CIWD secondary to ACS.

## Figures and Tables

**Table 1:** The clinical, demographic, and biochemical characteristics of patients with impaired and normal cognitive function.

Characteristic	All Patients (*n* = 50)	ICF (*n* = 22)	NCF (*n* = 28)	*p*-Value
Age, (years)	49.3 ± 7.24	48.04 ± 7.76	51.70 ± 6.46	0.384
Male, *n* (%)	27 (54)	11 (52)	16 (56)	0.531
Married, *n* (%)	44 (88)	19 (88)	25 (88)	1.000
Right-handed, *n* (%)	48 (96)	21 (96)	27 (96)	1.000
Educational level, *n* (%)				
- Middle school level	35 (70)	16 (72)	19 (70)	0.868
- High school level	15 (30)	6 (28)	9 (32)	0.516
History of parental kinship	7 (14)	3 (12)	4 (15)	0.624
History of family dementia	10 (20)	6 (25)	4 (16)	0.096
Heart rate (beat/minute)	75.10 ± 8.0	74.28 ± 8.21	75.92 ± 7.20	0.683
Systolic blood pressure (mmHg)	128.30 ± 10.2	126.96 ± 12.02	129.40 ± 11.34	0.085
Diastolic blood pressure (mmHg)	81.60 ± 8.20	80.20 ± 11.80	83.12 ± 8.18	0.073
BMI, kg/m^2^	27.10 ± 2.99	28.01 ± 2.24	27.90 ± 2.50	0.934
Glucose level (mg/dL)	112.32 ± 34.61	109.92 ± 30.50	114.72 ± 37.45	0.850
eGFR (mL/dk/1.73 m^2^)	99.5 ± 12.4	98.2 ± 12.8	99.8 ± 11.9	0.777
LDL-C (mg/dL)	122.02 ± 44.15	124.92 ± 42.09	120.12 ± 43.94	0.474
HDL-C (mg/dL)	40.00 ± 11.02	41.96 ± 12.78	38.04 ± 8.76	0.529
Total cholesterol (mg/dL)	170.0 ± 38.64	169.34 ± 37.49	170.56 ± 39.78	0.726
Current smoker, *n* (%)	41 (82)	18 (80)	23 (82)	0.868
STEMI, *n* (%)	25 (50)	13 (52)	12 (48)	0.352
Troponin I, ng/mL	42.62 ± 26.5	47.5 ± 26.2	34.8 ± 22.0	0.01 ^a^
AF, *n* (%)	0 (0)	0 (0)	0 (0)	1.00
LVEF, %	53.28 ± 3.8	52.15 ± 3.2	53.29 ± 3.5	0.538
Vitamin B12	270.96 ± 165.5	273.84 ± 160.45	271.08 ± 167.55	0.542
Folic acid	7.39 ± 2.60	7.27 ± 2.48	7.42 ± 2.77	0.998
Vitamin D	21.14 ± 8.84	20.48 ± 8.15	19.79 ± 8.51	0.794
Hospital stay, days	5.2 ± 0.8	5.3 ± 0.8	5.0 ± 0.7	0.437
PCI, *n* (%)	50 (100)	25 (100)	25 (100)	0.318
Medications at discharge				
- Acetylsalicylic acid, *n* (%)	50 (100)	25 (100)	25 (100)	
- P2Y12 inhibitors, *n* (%)	50 (100)	25 (100)	25 (100)	
- ACEI/ARB, *n* (%)	38 (76)	18 (72)	20 (80)	
- Statin, *n* (%)	50 (100)	25 (100)	25 (100)	
- Beta-blockers, *n* (%)	47 (94)	24 (96)	23 (92)	

Abbreviations: AF, atrial fibrillation; eGFR, estimated glomerular filtration rate; HDL-C, high-density lipoprotein cholesterol; ICF, impaired cognitive function; LDL-C, low-density lipoprotein cholesterol; LVEF, left ventricular ejection fraction; NCF, normal cognitive function; PCI, percutaneous coronary intervention; STEMI, ST elevation myocardial infarction. Data are expressed as mean ± SD or number of the patients (percentage). ^a^ patients with ICF vs. patients with NCF.

**Table 2:** The comparison of neurocognitive function tests and Beck Depression and Anxiety Inventory in patients with and without Fazekas on the first day and on the thirtieth day.

	Fazekas− (*n* = 25)			Fazekas+ (*n* = 25)		
	Day 1	Day 30	*p*-Value	Day 1	Day 30	*p*-Value
BDI	10.0 ± 10.0	8.60 ± 8.18	0.547	10.64 ± 8.12	11.08 ± 10.61	0.443
BAI	10.24 ± 9.72	9.32 ± 11.81	0.078	9.84 ± 7.93	7.88 ± 5.19	0.057
MMSE	26.80 ± 2.80	27.84 ± 2.56	0.001	27.24 ± 2.31	28.52 ± 1.78	0.003
DS forwards	5.96 ± 1.21	6.28 ± 1.37	0.410	6.32 ± 1.60	6.20 ± 1.38	0.768
DS backwards	3.60 ± 1.22	3.60 ± 1.22	1.000	3.68 ± 1.49	3.68 ± 0.90	0.916
Phonemic fluency	28.08 ± 16.76	34.36 ± 16.10	<0.001	30.28 ± 14.89	35.76 ± 14.23	<0.001
K	12.20 ± 6.52	13.60 ± 6.65	0.029	12.2 ± 6.19	14.24 ± 5.67	0.018
A	7.52 ± 4.52	10.24 ± 5.0	0.001	8.24 ± 5.13	10.44 ± 5.07	<0.001
S	8.36 ± 6.74	10.52 ± 5.77	0.001	9.84 ± 4.76	11.04 ± 5.11	0.037
BNT	23.32 ± 3.78	24.00 ± 3.15	0.025	23.56 ± 3.40	24.08 ± 3.04	0.007
Animal fluency	18.48 ± 5.46	20.64 ± 4.61	<0.001	17.84 ± 4.84	20.6 ± 4.05	0.004
Stroop test completion time	55.44 ± 29.57	48.08 ± 23.54	0.003	56.60 ± 25.15	49.24 ± 18.13	0.001
Stroop test error rate	7.96 ± 6.64	4.48 ± 5.72	<0.001	6.72 ± 7.14	4.08 ± 5.05	<0.001
CDT	3.60 ± 1.0	3.68 ± 0.85	0.317	3.56 ± 1.04	3.68 ± 0.90	0.083

Abbreviations: BAI, Beck Anxiety Inventory; BDI, Beck Depression Inventory; CDT, clock drawing test; DS, digit span; MMSE, Mini-Mental State Exam; BNT, Boston Naming Test. Data are expressed as mean ± SD.

**Table 3:** The comparison of neurocognitive function tests and Beck Depression and Anxiety Inventory in patients with and without MTLA on the first day and on the thirtieth day.

	MTLA− (*n* = 40)			MTLA+ (*n* = 10)		
	Day 1	Day 30	*p*-Value	Day 1	Day 30	*p*-Value
BDI	10.38 ± 9.43	9.85 ± 9.80	0.319	10.10 ± 7.64	9.80 ± 8.04	0.765
BAI	10.30 ± 9.45	8.50 ± 8.15	0.005	9.00 ± 5.91	9.00 ± 6.73	0.887
MMSE	27.18 ± 2.49	28.28 ± 2.23	<0.001	26.40 ± 2.84	27.80 ± 2.20	0.03
DS forwards	6.10 ± 1.43	6.18 ± 1.39	0.169	6.30 ± 1.42	6.50 ± 1.27	0.317
DS backwards	3.73 ± 1.41	3.68 ± 1.10	0.870	3.30 ± 1.06	3.50 ± 0.97	0.414
Phonemic fluency	28.90 ± 15.59	35.25 ± 14.35	0.005	30.30 ± 17.11	34.30 ± 18.45	0.024
K	12.15 ± 6.32	13.90 ± 5.85	0.002	12.4 ± 6.52	14.00 ± 7.47	0.237
A	7.70 ± 4.45	10.43 ± 4.84	0.043	8.60 ± 6.22	10.00 ± 5.81	0.07
S	9.05 ± 6.06	10.90 ± 5.34	<0.001	9.30 ± 5.03	10.30 ± 5.93	0.310
Animal fluency	18.35 ± 5.12	20.98 ± 4.02	0.118	17.40 ± 5.30	19.20 ± 5.25	0.150
BNT	23.48 ± 3.64	24.08 ± 3.13	0.003	23.30 ± 3.43	23.90 ± 2.42	0.08
Stroop test completion time	57.88 ± 25.95	49.58 ± 20.31	0.03	48.60 ± 32.04	45.00 ± 23.45	0.540
Stroop test error rate	7.40 ± 7.00	4.15 ± 4.02	0.02	7.10 ± 6.56	4.80 ± 4.65	0.04
CDT	3.60 ± 1.01	3.73 ± 0.82	0.06	3.50 ± 1.08	3.50 ± 1.08	1.00

Abbreviations: BAI, Beck Anxiety Inventory; BDI, Beck Depression Inventory; CDT, clock drawing test; DS, digit span; MMSE, Mini-Mental State Exam; BNT, Boston Naming Test. Data are expressed as mean ± SD.

**Table 4:** Test results for Fazekas groups.

Test	*t*-Test *p*-Value	Mann–Whitney U Test *p*-Value
BDI	0.9862937928807034	0.9816636125971189
BAI	0.9862937928807034	0.9816636125971189
MMSE	0.9862937928807034	0.9816636125971189
DS forwards	0.9862937928807034	0.9816636125971189
DS backwards	0.9862937928807034	0.9816636125971189
Phonemic fluency	0.9862937928807034	0.9816636125971189
Phonemic fluency K	0.9862937928807034	0.9816636125971189
Phonemic fluency A	0.9862937928807034	0.9816636125971189
Phonemic fluency S	0.9862937928807034	0.9816636125971189
Animal fluency	0.9862937928807034	0.9816636125971189
BNT	0.9862937928807034	0.9816636125971189
Stroop test completion time	0.9862937928807034	0.9816636125971189
Stroop test error rate	0.9862937928807034	0.9816636125971189
CDT	0.9862937928807034	0.9816636125971189

Abbreviations: BAI, Beck Anxiety Inventory; BDI, Beck Depression Inventory; BNT, Boston Naming Test; CDT, clock drawing test; DS, digit span; MMSE, Mini-Mental State Exam. Data are expressed as mean ± SD.

**Table 5:** Test results for MTLA groups.

Test	*t*-Test *p*-Value	Mann-Whitney U Test *p*-Value
BDI	0.8503914245106576	0.9633369080588674
BAI	0.8503914245106576	0.9633369080588674
MMSE	0.8503914245106576	0.9633369080588674
DS forwards	0.8503914245106576	0.9633369080588674
DS backwards	0.8503914245106576	0.9633369080588674
Phonemic fluency	0.8503914245106576	0.9633369080588674
Phonemic fluency K	0.8503914245106576	0.9633369080588674
Phonemic fluency A	0.8503914245106576	0.9633369080588674
Phonemic fluency S	0.8503914245106576	0.9633369080588674
Animal fluency	0.8503914245106576	0.9633369080588674
BNT	0.8503914245106576	0.9633369080588674
Stroop test completion time	0.8503914245106576	0.9633369080588674
Stroop test error rate	0.8503914245106576	0.9633369080588674
CDT	0.8503914245106576	0.9633369080588674

Abbreviations: MTLA, Medial Temporal Lobe Atrophy; BAI, Beck Anxiety Inventory; BDI, Beck Depression Inventory; BNT, Boston Naming Test; CDT, clock drawing test; DS, digit span; MMSE, Mini-Mental State Exam. Data are expressed as mean ± SD.

## Data Availability

Data is contained within the article.
